# Resistance to DNA Damaging Agents Produced Invasive Phenotype of Rat Glioma Cells—Characterization of a New in Vivo Model

**DOI:** 10.3390/molecules21070843

**Published:** 2016-06-27

**Authors:** Sonja Stojković, Ana Podolski-Renić, Jelena Dinić, Željko Pavković, Jose M. Ayuso, Luis J. Fernández, Ignacio Ochoa, Victor M. Pérez-García, Vesna Pešić, Milica Pešić

**Affiliations:** 1 Department of Neurobiology, Institute for Biological Research “Siniša Stanković”, University of Belgrade, Despota Stefana 142, Belgrade 11060, Serbia; sonja.stojkovic@ibiss.bg.ac.rs (S.S.); ana.podolski@ibiss.bg.ac.rs (A.P.-R.); jelena.dinic@ibiss.bg.ac.rs (J.D.); zeljko.pavkovic@ibiss.bg.ac.rs (Ž.P.); vesnav@ibiss.bg.ac.rs (V.P.); 2 Group of Structural Mechanics and Materials Modeling (GEMM), Centro Investigacion Biomedica en Red. Bioingenieria, Biomateriales y Nanomedicina (CIBER-BBN), Zaragoza, Aragon 50018, Spain; josayuso@unizar.es (J.M.A.); luisf@unizar.es (L.J.F.); iochgar@unizar.es (I.O.); 3 Aragón Institute of Engineering Research (I3A), University of Zaragoza, Zaragoza, Aragon 50018, Spain; 4 Aragon Institute of Biomedical Research, Instituto de Salud Carlos III, Madrid, Madrid 28029, Spain; 5 Departamento de Matemáticas, E.T.S.I. Industriales and Instituto de Matemática Aplicada a la Ciencia y la Ingeniería (IMACI), Universidad de Castilla-La Mancha, Ciudad Real 13071, Spain; Victor.PerezGarcia@uclm.es

**Keywords:** antiglioma therapy, chemoresistance, DNA damaging agents, glioma, invasion, in vivo model, behavior study

## Abstract

Chemoresistance and invasion properties are severe limitations to efficient glioma therapy. Therefore, development of glioma in vivo models that more accurately resemble the situation observed in patients emerges. Previously, we established RC6 rat glioma cell line resistant to DNA damaging agents including antiglioma approved therapies such as 3-bis(2-chloroethyl)-1-nitrosourea (BCNU) and temozolomide (TMZ). Herein, we evaluated the invasiveness of RC6 cells in vitro and in a new orthotopic animal model. For comparison, we used C6 cells from which RC6 cells originated. Differences in cell growth properties were assessed by real-time cell analyzer. Cells’ invasive potential in vitro was studied in fluorescently labeled gelatin and by formation of multicellular spheroids in hydrogel. For animal studies, fluorescently labeled cells were inoculated into adult male Wistar rat brains. Consecutive coronal and sagittal brain sections were analyzed 10 and 25 days post-inoculation, while rats’ behavior was recorded during three days in the open field test starting from 25th day post-inoculation. We demonstrated that development of chemoresistance induced invasive phenotype of RC6 cells with significant behavioral impediments implying usefulness of orthotopic RC6 glioma allograft in preclinical studies for the examination of new approaches to counteract both chemoresistance and invasion of glioma cells.

## 1. Introduction

The most abundant and the most aggressive form of primary brain tumors is glioblastoma multiforme (GBM). GBM main features are invasiveness and resistance to radio- and chemotherapy that lead to patients’ worse prognosis [1]. The prognosis for the 5-year survival rate of GBM patients is less than 9.8% [2]. The major barriers to the successful GBM treatment are development of resistance mechanisms and the ability of GBM cells to rapidly invade the brain and disrupt the architecture of brain parenchyma [3].

Despite the enormous knowledge about GBM genesis and progression, the clinical implementation of new therapeutics has not significantly progressed. As an illustration to this is the fact that only three of all FDA-approved oncology drugs are regularly used to treat GBM: biodegradable carmustine—bis-chloroethylnitrosourea (BCNU) wafers (Gliadel; FDA approval in 1995), temozolomide (TMZ; Temodar; FDA approval in 1997), and bevacizumab (Avastin; FDA approval in 2009 for recurrent GBM). The current treatment for GBM patients consists of multimodal approach including maximal safe tumor resection followed by radiotherapy and simultaneous daily TMZ followed by adjuvant TMZ according to Stupp’s protocol [4]. Despite surgical removal and treatment, diffuse infiltrative growth of the remaining GBM cells confer to tumor recurrence and patients’ death within 1–2 years of diagnosis [5].

C6 rat glioma model is one of the most extensively used glioma models for the evaluation of the efficacy of different chemotherapeutic approaches. It was established by continuous application of methyl nitrosourea (MNU) to outbred Wistar rats for approximately 8 month [6]. Molecular characterization of C6 glioma cells revealed many similarities between the gene expression pattern observed in these cells and that reported in human GBMs [7]. C6 glioma is a suitable model for the invasion studies due to the presence of pleomorphic cells with characteristic focal invasion into brain parenchyma [6]. Although other authors showed the presence of chemoresistant side population cells in C6 glioma [8], so far this model was not sufficiently employed for the examination of drug resistance and strategies for its overcoming.

In our previous work, we established resistant RC6 rat glioma cell line from its corresponding parental C6 cell line. The resistance was induced by continuous application of BCNU, a DNA alkylating agent. Characterization of RC6 phenotype showed cross-resistance to other DNA damaging agents including TMZ [9]. The major mechanism of resistance in RC6 cells provoked by continuous BCNU treatment was the adaptation to oxidative stress shown by elevated level of reactive oxygen species (ROS), increment of glutathione detoxification and a significant increase in antioxidant enzymes’ (iNOS, MnSOD and GPx) expression [9].

Due to high basal metabolic rate, GBM cells readily produce ROS as natural by-products of aerobic metabolism [10]. ROS impacts some of the most important mechanisms for GBM cell survival such as proliferation, angiogenesis and invasion [11,12]. Particularly, high level of MnSOD is associated with worse prognosis in GBM [13], while numerous studies indicate high ROS content as a driving force for cancer cells’ migration and invasion [14,15].

We suspect that ROS plays central role in both, GBM resistance to therapy and invasion properties. Therefore, we used RC6 rat resistant glioma cell line adapted to high ROS content [9] to evaluate its invasiveness in 3D cell culture and orthotopic allograft animal model in Wistar rats. As a specific aim of this study, we asserted the comparison between C6 and RC6 animal models. Many different studies showed that the rat C6 glioma model can be used to investigate different therapeutic modalities [16,17]. However, there is a necessity for the development of experimental animal models that resemble in an appropriate manner the clinical reality observed in GBM patients. In this study, we characterize new rat glioma animal model with RC6 cells which could be further utilized for the development of new therapeutic strategies oriented to combat both, GBM resistance and invasion. To that end, we investigated and compared the invasion properties of C6 and RC6 cells in vitro and through brain parenchyma as well as the influence on rats’ behavior that could be affected by the presence of glioma cells in the brain.

## 2. Results

### 2.1. Difference in the Proliferation Rate of C6 and RC6 Cells

**Figure 1:**
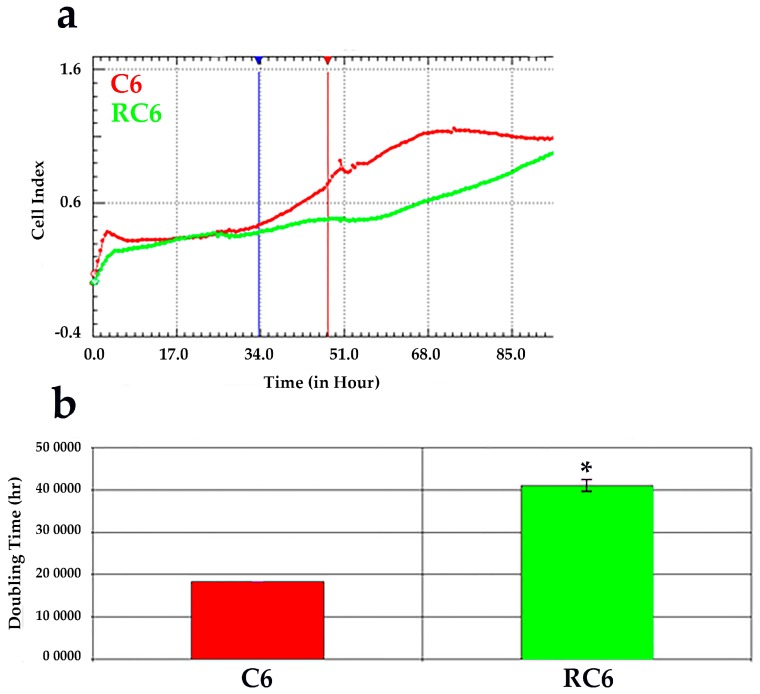
Real time analysis of proliferation of C6 and RC6 cells using E-plate. The rate of proliferation was monitored using the xCELLigence system. (**a**) Representative graph comparing the Cell Index (CI) of C6 (**red line**) and RC6 cell line (**green line**); (**b**) Doubling time of C6 and RC6 cell line determined by analyzing the cell growth in logarithmic phase between 34 h and 48 h time points. The average ± SEM obtained from three independent experiments (*n* = 3) are presented. Statistical significance is expressed as *p* < 0.05 (*).

The proliferation rate of C6 and RC6 cells was analyzed in real-time using the xCELLligence system. C6 and RC6 cells were seeded in E-plate and their proliferation was monitored over a period of 96 h. The proliferation rate was expressed as Cell Index (CI) and doubling time of C6 and RC6 cells was calculated by analyzing the line slope between time points that corresponds to logarithmic cell growth (Figure 1a). Results indicate that C6 cells proliferate significantly faster (~2 fold) than RC6 cells The doubling time for C6 cells was ~20 h, while RC6 cells duplicate after ~40 h (Figure 1b, * *p* = 0.04).

### 2.2. Invasive Properties of C6 and RC6 Cells in Vitro

**Figure 2:**
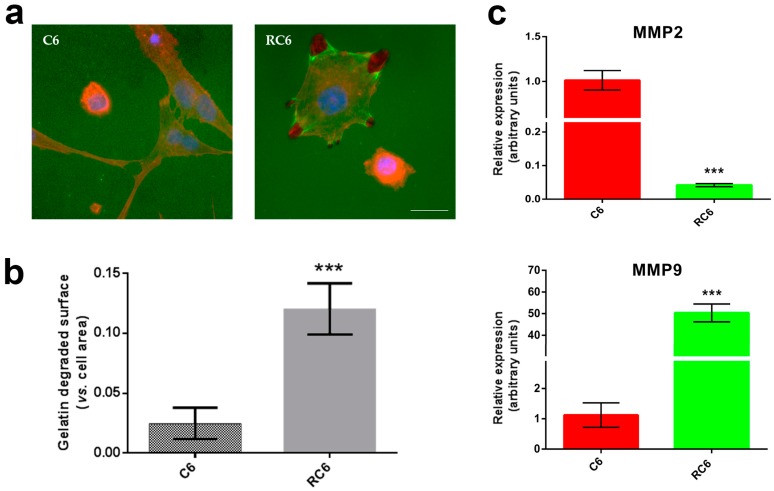
Gelatin degradation activity of C6 and RC6 cells. (**a**) Cells were plated on Oregon Green^®^ 488 Conjugate Gelatin for 24 h and afterwards stained with Hoechst 33342 (**blue**) and ActinRed 555. Areas of gelatin degradation appear as dark spots in the fluorescent background beneath the cells. Scale bar = 200 μm; (**b**) Quantification of degraded surface of Oregon Green^®^ 488 Conjugate Gelatin per total area of cells. The average ± SEM obtained from three independent experiments (*n* = 3) are presented. Statistical significance is shown as *p* < 0.001 (***); (**c**) Real-time qRT-PCR analysis was applied to assess MMP2 and MMP9 mRNA expression. As an internal control, β-actin mRNA expression was used (*n* = 3). Statistical significance is presented as *p* < 0.001 (***).

**Figure 3:**
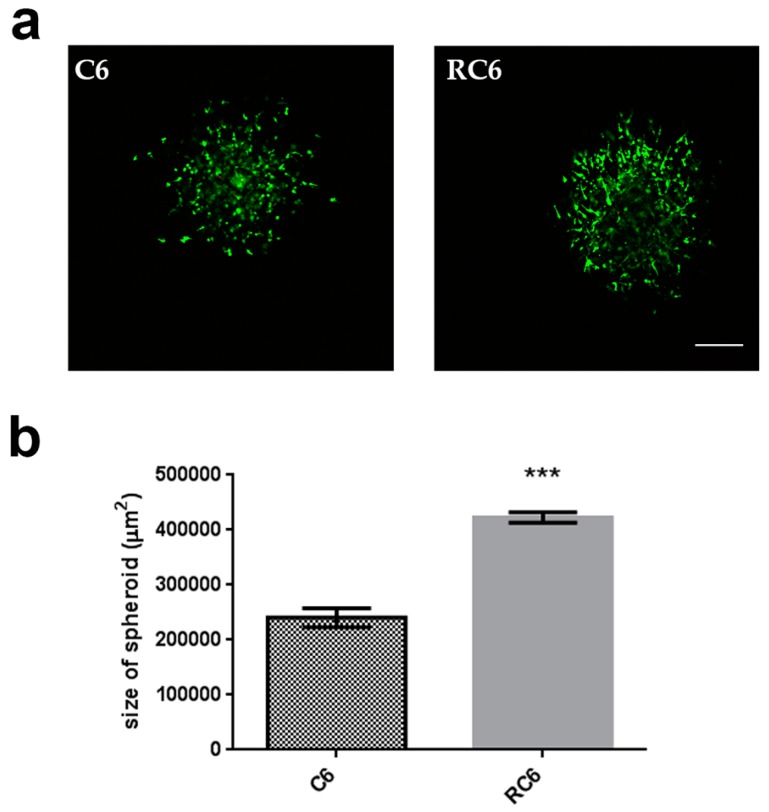
3D invasion of spheroids formed from C6 and RC6 cells. (**a**) Representative images of C6 and RC6 spheroids embedded in hydrogel for 24 h and afterwards stained with CAM (**green**) and PI (**red**), Scale bar = 200 μm; (**b**) Invasion was quantified as size of spheroids 24 h after embedding in hydrogel. The average ± SEM obtained from three independent experiments (*n* = 3) are presented. Statistical significance is shown as *p* < 0.001 (***).

Invasive capacity of C6 and RC6 cells was evaluated in vitro by two assays: gelatin degradation (Figure 2) and 3D spheroid invasion (Figure 3).

The proteolytic activity of C6 and RC6 cells was analyzed using Oregon Green^®^ 488 Conjugate gelatin (Figure 2). The appearance of dark areas lacking fluorescence indicates the proteolysis of fluorescent gelatin. As shown in Figure 2a degraded areas were scarcely detected in the C6 cells whereas the presence of RC6 cells significantly increased the areas of degradation (~4 fold) compared to C6 (Figure 2b, *** *p* = 0.0007). Moreover, we demonstrated that MMP9 mRNA expression in RC6 cells overcomes the expression in C6 cells by 50 fold (Figure 2c, *** *p* = 0.0003) while the expression of MMP2 mRNA in RC6 cells is significantly decreased by 25 fold (Figure 2c, *** *p* = 0.0009).

Next, the invasion of C6 and RC6 spheroids was assessed in 3D hydrogel (Figure 3). Both cell lines formed well defined uniform spheroids after 24 h using hanging drop method. Afterwards, spheroids were embedded in hydrogel for 24 h, and then stained with fluorescent dyes CAM and PI (Figure 3a). The fraction of cells invading hydrogel is expressed as a size of spheroids. Invasion of RC6 spheroids was significantly increased (~2 fold) compared to that of C6 spheroids (Figure 3b, *** *p* = 0.0007).

### 2.3. Invasive Properties of C6 and RC6 Cells in Vivo

**Figure 4:**
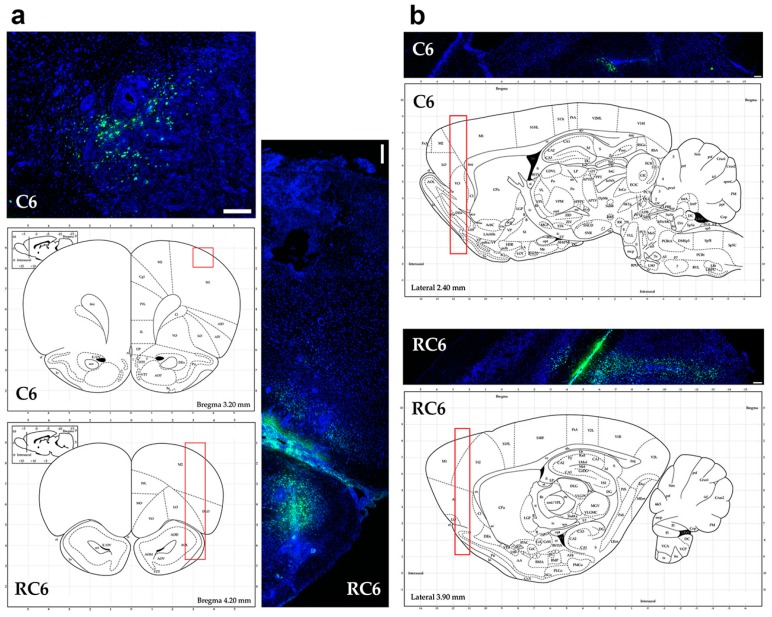
Invasion of C6 and RC6 cells 10 days after inoculation. (**a**) Representative images of coronal; (**b**) and sagittal sections of Wistar rat brain from C6 and RC6 group. Inoculated cells were stained with fluorescent dye FB (**green**). Nuclei were counterstained with PI (**blue**). Scale bar = 200 μm. Rat brain schemes were taken from Rat Brain Atlas [18].

**Figure 5:**
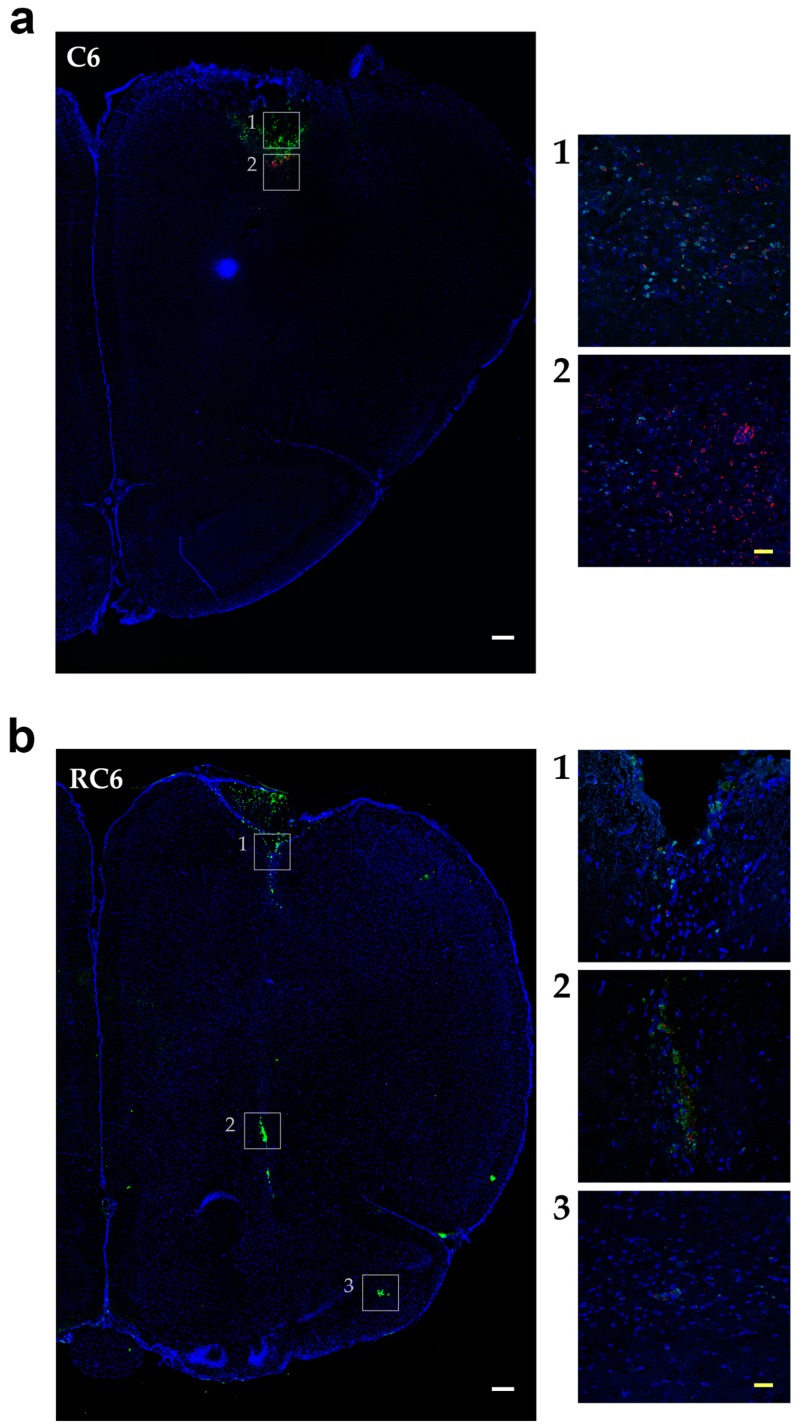
Invasion of C6 and RC6 cells 25 days after inoculation. (**a**) Representative images of coronal sections of Wistar rat brain from C6; and (**b**) RC6 group. Inoculated cells were stained with fluorescent dye CFSE (**green**) and proliferation marker anti-Ki67 antibody (**red**). Nuclei were counterstained with Hoechst 33342 (**blue**). Scale bar = 200 μm (**white**), scale bar = 25 μm (**yellow**).

Observation of the glioma cell invading properties in animal model was enabled by inoculation of FB (Figure 4) or CFSE (Figure 5) fluorescently labeled C6 and RC6 cells in the Wistar rats’ brain. The health of animals was monitored on a daily basis.

FB fluorescent dye was used to track C6 and RC6 cells 10 days post-inoculation. C6 cells were mostly found near the injection site (Figure 4a, coronal section) with a small potential for penetration into the deeper layers of brain (Figure 4b, sagittal section). Clusters of C6 cells had unclear demarcation line which coincided with infiltrative spread of these cells in vivo. Unlike C6 cells, RC6 cells migrated deeper into the brain parenchyma (Figure 4a, coronal section). The fluorescent signal originating from the RC6 cell was more intense than the signal detected from inoculated C6 cells. RC6 cells were found in ipsilateral olfactory bulb and in the transition to the olfactory bulb (Figure 4b, sagittal section).

CFSE fluorescent dye was used to follow the invasion of C6 and RC6 cells 25 days post-inoculation. The ability of CFSE to emit detectable fluorescence persists longer—several cell divisions more than in the case of FB fluorescent dye. Ki-67 was used for colocalization study as a universal proliferating marker which expression can be found in G1, S, G2, and mitosis, but not in the G0 resting cells. Invasion pattern of C6 cells was similar to that observed 10 days after inoculation. Ki-67 positive cells colocalized with CFSE stained cells near the site of inoculation where the invasive front was formed (Figure 5a, coronal section). RC6 cells infiltrated distant ipsilateral brain structures. CFSE stained RC6 cells colocalized with Ki67 positive cells in different brain regions (Figure 5b, coronal section).

### 2.4. Motor Activity and Habituation of Experimental Animals with C6 and RC6 Cells

**Figure 6:**
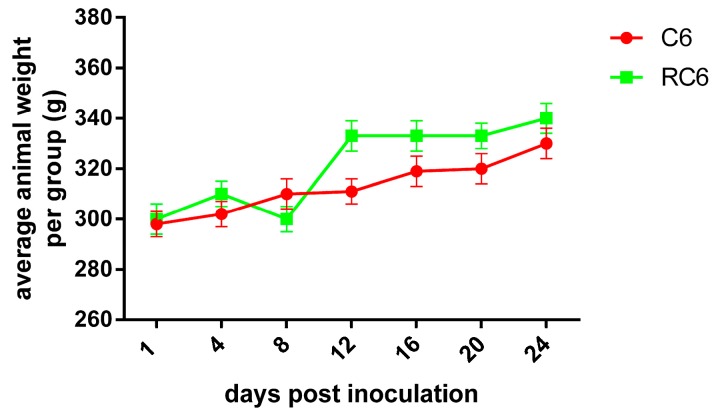
Average animal weight in C6 and RC6 group followed during 24 days after cells’ inoculation.

Behavior studies were performed with animals that did not suffer according to the physiological parameters as their average body weight was not significantly changed. Even more, a general trend of weight gaining in rats with inoculated C6 and RC6 cells was observed during 24 days (Figure 6).

**Figure 7:**
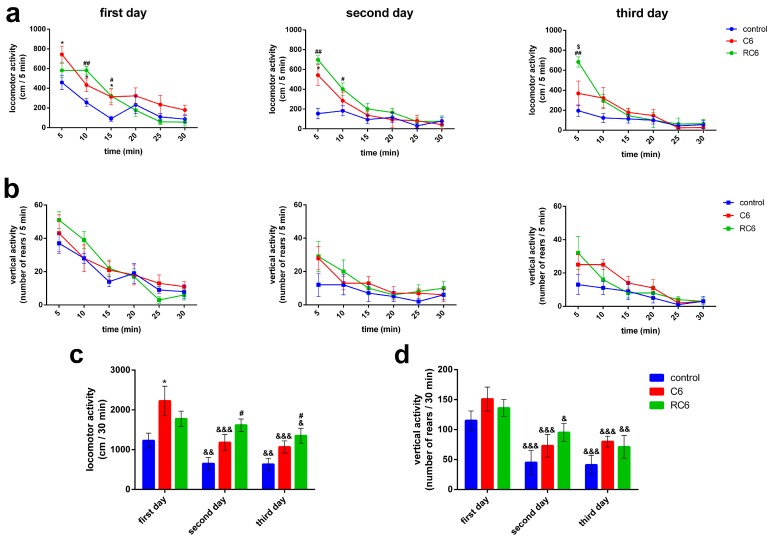
Motor activity and habituation of control, C6 and RC6 groups of the animals. (**a**) 5 min scores of locomotor activity; (**b**) and vertical activity of animals during 30 min period (intra-session habituation), across 3 consecutive days of registration; (**c**) Total locomotor activity; (**d**) and vertical activity of animals for the 30 min registration period during 3 consecutive days (inter-session habituation). *p* < 0.05 (*) C6 vs. control group; *p* < 0.05 (#) and *p* < 0.01 (##) RC6 vs. control group; *p* < 0.05 ($) RC6 vs. C6 group; *p* < 0.05 (&), *p* < 0.01 (&&) and *p* < 0.001 (&&&) second/third day vs. the first day of the same experimental group.

All tested groups of animals had intra-session habituation, as manifested through decrease in locomotor (Figure 7a) and vertical (Figure 7b) activities during the registration period. Compared to the control group, C6 group had significantly higher locomotor activity at the first day of testing during the first 15 min of registration (Figure 7a, * *p* = 0.043, U test) as well as at the second day of testing during the first 5 min of registration (* *p* = 0.011). Compared to the control group, RC6 group had significantly higher locomotor activity at the first day of testing during the second (Figure 7a, ## *p* = 0.004) and the third 5 min of registration (# *p* = 0.045), at the second day of testing during the first (## *p* = 0.004) and the second 5 min of registration (# *p* = 0.037) and at the third day of testing during the first 5 min of registration (## *p* = 0.004). Moreover, at the third day of testing RC6 group had significantly higher locomotor activity during the first 5 min of registration when compared to C6 group ($ *p* = 0.028). Importantly, U test did not reveal significant differences between the control, C6 and RC6 groups of rats regarding 5 min scores for vertical activity (Figure 7b).

Inter-session habituation is detected in all tested groups of animals regarding locomotor (Figure 7c) activity, as manifested through decrease in measured parameter across 3 days of registration. Statistical analysis revealed significant influence of the type of cell line inoculated (F (2, 14) = 6.295, *p* = 0.011), time (F (2, 28) = 24.339, *p* < 0.001) and treatment x time interaction (F (2, 28) = 3.134, *p* = 0.031). Within-group comparisons revealed that measured parameter was decreased during the second and the third day of registration in comparison to the first day in the control group (&& *p* = 0.004 and && *p* = 0.003, respectively) and C6 group (&&& *p* < 0.001), while in RC6 group significant decrease was achieved only at the third day of registration (& *p* = 0.031). In comparison to the control group locomotor activity of C6 group was significantly higher during the first day of testing (* *p* = 0.029), while the activity of RC6 group was significantly higher during the second (# *p* = 0.027) and the third (# *p* = 0.045) day. Other comparisons did not reveal significant differences.

Changes in vertical activity of experimental animals across 3 days of registration are presented in Figure 7d. Statistical analysis revealed significant influence of time (F (2, 28) = 25.831, *p* < 0.001) and insignificant influence of the type of inoculated cell line (F (2, 14) = 2.423, *p* = 0.128) and treatment x time interaction (F (2, 28) = 0.603, *p* = 0.664). Compared to the locomotor activity achieved at the first day of registration measured parameter was decreased during the second and the third day of registration in the control group (&&& *p* < 0.001), C6 group (&&& *p* < 0.001) and RC6 group (& *p* = 0.041 and && *p* = 0.002, respectively). Comparison between groups did not reveal significant differences at any of testing days.

## 3. Discussion

Modern therapy of GBM is challenged by two key features: drug resistance and dissemination of cancer cells that both prevent efficient therapy. The ability of GBM cells to evade and resist chemo- and radiotherapy is due to phenotypic changes, from proliferative to migratory state. In addition, invasion of GBM cells into the surrounding brain parenchyma is considered as a reason for surgical resection failure because even after extensive elimination of almost 99% of the tumor mass, average recurrence of GBM is in less than six months [19]. Herein, we investigated the association between chemoresistant phenotype of recently characterized RC6 resistant rat glioma cells [9] and their invasion properties in vitro and in vivo. As a model for comparative study, we used C6 cells from which RC6 originated after induction of resistance to BCNU and other DNA damaging agents.

The main mechanisms of chemoresistance recognized in RC6 cells include adaptation to high levels of ROS due to increased expression of intracellular antioxidants such as MnSOD and iNOS as well as the engagement of glutathione detoxification system with increased expression of GPx and MRP1 [9]. Under continuous pressure of BCNU treatment during resistance induction, C6 cells were exposed to DNA damage and oxidative stress caused by dramatically increased ROS production. As an attempt to restore redox balance, C6 cells increased the expression of antioxidant enzymes. Thus, the presence of ROS provoked adaptive response enabling the survival of RC6 cells with a new phenotype. By lowering oxidative stress, RC6 cells potentially produced switch from highly proliferative C6 to more invasive RC6 phenotype. Increased intracellular ROS concentration activates the matrix metalloproteinases MMP2 and MMP9 leading to extracellular matrix degradation and eventually migration of cells [20]. In addition, resistant phenotype is usually connected with stemness characteristics of cancer cells and lower proliferation rate [21].

To evaluate the changes in cell growth characteristics between C6 and RC6 cells, we employed the analysis of cell growth in real-time using impedance measurement equivalent to cell growth index. According to obtained time of duplication, RC6 cells grow significantly slower than C6 cells and this may imply the dedifferentiation of C6 cells to a new phenotype with stemness characteristics. In addition, development of chemoresistance in RC6 cells could produce the switch toward more invasive phenotype. Knowing that invasive cell phenotype possesses increased cellular motility and greater activation of matrix metalloproteinases that both lead to degradation of extracellular matrix [22], we tested the potential of C6 and RC6 cells to degrade gelatin in vitro. The extent of degraded gelatin is a consequence of the activity of MMP2 and MMP9 [23]. We found that RC6 cells more readily degrade gelatin than C6 cells suggesting the potential to demonstrate the same behavior in vivo. The increased ability of RC6 cells to degrade gelatin could be related to the significant increase in the MMP9 mRNA expression. Then, we drew our attention to the examination of cell invasion in 3D in vivo mimicking conditions. Both C6 and RC6 cells formed spheroids which were afterwards embedded in hydrogel. The size of spheroids that corresponds to invading distance in hydrogel was assessed and RC6 cells showed greater potential to invade hydrogel as their spheroids were significantly larger than C6 spheroids.

However, in vitro conditions do not resemble accurately the situation in vivo. Primary brain tumors including GBM possess the unique patterns of invasion and metastasis mainly within CNS [24]. This suggests that these tumors are adapted to the specific microenvironment present in CNS. In addition, it is known that the extracellular environment modulates angiogenesis and cell invasion, and may control tumor growth and spread independently of the cancer clone phenotype [25]. Therefore, we examined the differences in invasion properties of C6 and RC6 cells in vivo using orthotopic allograft model in Wistar rats.

There are evidences that C6 cells show the pseudopalisade cellular formation in vivo [26,27]. Brat and Van Meir [28] demonstrated that proliferation rates are lower in pseudopalisades than in adjacent tumor tissue. In addition, GBM invasion increases if coincides with pseudopalisades development [28]. Considering these findings, we assumed that both C6 and RC6 cells will efficiently invade the brain parenchyma.

Tracing the C6 and RC6 cells by vital fluorescent dyes in Wistar rat brain showed considerable differences in invasion pattern of these two cell types. C6 cells were predominantly found near inoculation site after 10 and 25 days post-inoculation, while RC6 cells migrated toward olfactory bulb and other distant structures. Significant limitation of C6 and RC6 in vivo model is infiltratory cell growth that is hard to follow. We expected that C6 cells could also migrate but due to their high proliferative capacity the tracking fluorescence vanished during cell divisions. Therefore, we used proliferation marker Ki67 to localize C6 and RC6 cells more precisely. By colocaliziation with Ki67, we were able to confirm that trajectory of C6 and RC6 migration 25th day post-inoculation corresponds to that observed by fluorescence tracer. C6 cells were found near inoculation site, while RC6 cells showed distant ipsilateral infiltration. Considering that de novo neurogenesis in the olfactory bulbs of various adult rodent brains was observed [29], we cannot exclude the possibility that RC6 cells migratory path was directed by chemoattractants produced by neural stem cells present in olfactory bulbs.

Behavioral examinations pointed to the peculiar locomotor activity of rats with inoculated C6 and RC6 cells, as assessed 25 days after inoculation of the cells. The vertical activity was not compromised in both C6 and RC6 groups. Importantly, as control group received appropriate volume of vehicle, any doubt about the influence of the experimental procedure per se was denied. Compared to the control group, rats inoculated with C6 cells showed increase in locomotor activity during the exposure to the arena on the first day of testing, reflecting the treatment-related hyperactivity. Although RC6 rats also showed increased locomotor response during the first 10 min of testing at the first day this increase was not evident by the total score. Since the brain region of interest where the cells were inoculated in our study was primary motor cortex we assume that changes in its functioning, in terms of sensory-motor integration and control of the movements, contributed to the observed behavioral outcome of the treatment. Relationship between exploratory behavior and the activity of the primary somatosensory and the motor cortex has been shown, together with selective increase in the activity of the hippocampus [30]. Importantly, in C6 group of rats the signs of disturbed habituation were subtly evident during the second day of testing while in RC6 group of rats they were robustly evident during both the second and third day of testing. These results pointed that in C6 group of rats the ability to recognize already explored environment was slightly disturbed, while more pronounced disturbances in the recognition/adaptability to the novel environment was present in RC6 group of rats. In general, these findings support the role of motor cortex in learning. Previous findings already accentuated its function in the connection between movement and sensation underlying complex learned behaviors [31] as well as the role of correct motor cortical processing during the action in later recognition [32]. As the activity of the hippocampus is important in habituational learning [33], secondary dysfunction of the hippocampal projections to the cortex should not been excluded and thus remained to be examined in further studies. Nevertheless, our experiments undoubtedly showed that compared to C6 cells, RC6 cells more invasively disturbed neural substrates/dynamic interactions important for multimodal processing of informations and subsequent adaptation to the novelty, learning and memory.

In conclusion, many characteristics of RC6 are related to stem phenotype including chemoresistance, slow growth, capability for spheroids formation and invasiveness [34]. Further examinations are required to confirm the stemness of RC6 such as ability for self-renewal, propagation of phenotypically similar tumors upon secondary transplantation in animals and potential to differentiate into neurons and glia-like progenies. Nevertheless, RC6 cells are considerably resistant to TMZ (the main chemotherapy for GBM patients) and their orthotopic allograft model could be used as a valuable preclinical tool for the examination of the efficacy of new antiglioma therapies. The therapeutic window of 25 days during which fluorescently labeled RC6 cells could be traced inside the brain is sufficient for the evaluation of therapeutics’ potential to sensitize glioma cells, suppress invasion and improve behavior of rats with injected RC6 cells. In future, the examination by magnet resonance imaging would significantly contribute to our postulated goal: to use RC6 in vivo model as a preclinical tool for studying new strategies able to overcome both glioma chemoresistance and invasion.

## 4. Materials and Methods

### 4.1. Chemicals and Reagents

DMEM medium, l-glutamine and trypsin/EDTA were purchased from Biological Industries, Israel. Fetal bovine serum (FBS), dimetyl sulfoxide (DMSO), methylcellulose, Fast Blue (FB), Propidium Iodide (PI) and Hoechst 33342 were obtained from Sigma-Aldrich Chemie Gmbh, Darmstadt, Germany. TritonTM X-100 was purchased from Merck KgaA, Darmstadt, Germany. Calcein (CAM) was obtained from Thermo Fisher Scientific, Waltham, MA, USA. Collagen type I was purchased from Corning^®^, New York, NY, USA. Carboxyfluorescein succinimidyl ester (CFSE) was obtained from Molecular Probes^®^, Invitrogen, Waltham, MA, USA. Nembutal anesthetic was obtained from Serva, Heidelberg, Germany. Ki-67 Rabbit mAb was purchased from Cell Signaling Technology, Danvers, MA, USA. Secondary antibody Alexa Fluor 555 goat anti-rabbit IgG (H + L) and ActinRed 555 were obtained from Invitrogen Life Technologies, Waltham, MA, USA.

### 4.2. Cell Lines and Cell Culture

C6 cell line was purchased from American Type Culture Collection, Manassas, VA, USA. RC6 cells were selected from C6 cells after 9 months of BCNU selective pressure [9]. Both cell lines were cultured in DMEM medium supplemented with 10% FBS, 2 mM l-glutamine, 4.5 g/L glucose, 5000 U/mL penicillin and 5 mg/mL streptomycin solution at 37 °C in humidified 5% CO_2_ atmosphere. Cell lines were subcultured in 72 h intervals using 0.25% trypsin/EDTA and seeded into fresh medium in a concentration of 8000 cell/cm^2^.

### 4.3. Cell Proliferation RTCA Assay

Cell proliferation of C6 and RC6 cells was monitored in real-time using the xCELLigence system (Roche, Germany) with 96-well E-plate. Before seeding cells in E-plate, a standard background was measured by adding 50 μL of media at 37 °C to wells. Then, 4000 cells/well were seeded and the total volume of wells was adjusted to 200 μL with media. The impedance value of each well was automatically monitored by the xCELLigence system every 30 min for duration of 96 h and expressed as a CI (cell index) value. The rate of cell growth was determined by calculating the line slope between two given time points.

### 4.4. Gelatin Degradation Analysis

C6 and RC6 cells were plated in 6-well plates with glass coverslips coated with AlexaFluor488 labeled gelatin (Gelatin from Pig Skin, Oregon Green^®^ 488 Conjugate, Life Technologies, Waltham, MA, USA). After 24 h, cells were fixed with 4% paraformaldehyde and co-stained with Hoechst 33342 and ActinRed 555. Cells and degradation area were analyzed under a Zeiss Axiovert inverted fluorescent microscope (Carl Zeiss Foundation, Heidenheim, Germany). Volume of the dark area caused by degradation of gelatin was measured in ImageJ software (1.48, Microsoft, Redmond, WA, USA) and normalized in relation to the volume of the cell. At least 100 cells were analyzed per experiment.

### 4.5. RNA Extraction and Real-Time Quantitative PCR

Total RNA was isolated from C6 and RC6 cell lines. The isolation was carried out using Trizol^®^ reagent (Invitrogen Life Technologies) according to the manufacturer’s instructions. RNA was quantified by spectrophotometry and quality was determined by agarose gel electrophoresis. RT reactions were performed using 2 μg of total RNA, with a high-capacity cDNA reverse transcription kit (Applied Biosystems, Carlsbad, CA, USA), following the manufacturer’s instructions. In order to determine MMP2 (forward primer 5′-TTC TTC GCA GGG AAT GAG-3′; reverse primer 5′-ACG ACA GCA TCC AGG TTA T-3′) and MMP9 (forward primer 5′-AAA TGT GGG TGT ACA CAG GC-3′; reverse primer 5′-TTC ACC CGG TTG TGG AAA CT-3′) mRNA expression level in C6 and RC6 cells, real time PCR was performed. Prepared cDNAs were amplified using Maxima SYBR Green/ROX qPCR Master Mix (Thermo Scientific, Waltham, MA, USA), according to the recommendations of the manufacturer, in a total volume of 20 μL in a QuantStudio 3 Real-Time PCR System (Thermo Scientific). Thermocycler conditions comprised an initial step at 50 °C for 5 min, followed by a step at 95 °C for 10 min and a subsequent 2-step PCR program at 95 °C for 15 s and 60 °C for 60 s for 40 cycles. The accumulation of PCR products was detected in real time and the results were analyzed using the QuantStudio™ Design and Analysis Software 1.3.1. (Thermo Scientific) and presented as 2−ΔCt [35], ΔCt being the difference between Ct values of specific genes and the endogenous control (β-actin).

### 4.6. Spheroid Generation and 3D Tumor Spheroid Invasion Assay

C6 and RC6 multicellular spheroids were generated using hanging drop method with methylcellulose [36]. C6 and RC6 cell suspension was mixed with methylcellulose solution and 25 μL droplets (500 cell/droplet) were placed on the top of Petri dishes, sterile water was placed on the bottom to prevent droplet evaporation. After 24 h each droplet contained single well-defined spheroid.

For 3D invasion assay 25 μL of spheroids were imbedded in hydrogel mixture (17.85 μL collagen type I (4.01 mg/mL stock solution); 0.45 μL 1 M NaOH; 5 μL 5 × DMEM; 1.7 μL dH_2_O) and placed on top of 50 μL of hydrogel without spheroids. After 24 h spheroids were stained with CAM (1:1000, 5 mg/mL stock solution) and PI (1:500, 2 mg/mL stock solution) to test cell viability.

Images of spheroids were taken using a Nikon Eclipse Ti microscope (Nikon Instruments Inc., Tokyo, Japan) equipped with a C1 modular confocal microscope system. Spheroid invasion was analyzed using Fiji^®^ software for Nikon images (ND2). At least 10 spheroids were analyzed per experiment.

### 4.7. Labeling of C6 and RC6 Cells with FB and CFSE

FB and CFSE dyes are widely used for fluorescent labeling of cells. FB incorporates in cytoplasm of cells without affecting their viability and its presence can be detected over the period of 10 cell divisions [37]. In animal experiments FB dye was used for in vivo tracking of C6 and RC6 cells 10 days after inoculation. For FB labeling, cells were incubated for 10 min in DMEM containing FB diluted 1:100, made from 10 mg/mL stock solution. Incubation was carried out at room temperature, in dark. After incubation, cells were washed three times with PBS.

CFSE dye binds covalently to all free amines on the surface and inside of cells with minimal observed effect on the proliferative ability of cells. This dye enables monitoring of the cells over a period of 15 cell divisions [38]. Therefore, it was used for in vivo tracing of C6 and RC6 cells 25 days after inoculation. For CFSE labeling, cells were incubated for 10 min in 0.1% FBS/PBS solution containing 10 μM CFSE. Incubation was carried out at room temperature, in dark. After incubation, cells were washed three times with PBS.

### 4.8. Animals

At the beginning of the experiment, adult male Wistar rats were 3 months old and weighted 250–300 g. The animals were housed under standard conditions (23 ± 2 °C, 60%–70% relative humidity, 12 h light and dark cycles, with the lights switched on at 07:00, free access to water and food, *n* = 3 per cage). All animal procedures were in compliance with Directive (2010/63/EU) on the protection of animals used for experimental and other scientific purposes and was approved by the Ethical Committee for the Use of Laboratory Animals of the Institute for Biological Research “Siniša Stanković”, University of Belgrade. Minimal number of animals was used and all efforts were made to minimize animal suffering.

### 4.9. Intracranial Inoculation of C6 and RC6 Cells

The animals were anesthetized with intraperitoneal injection of Nembutal at a dose of 50 mg/kg body weight. A burr hole was made 3 mm lateral from the midline and 3 mm anterior of the Bregma. 105 of fluorescently labeled C6 or RC6 cells in 5 μL suspension was injected at the depth of 2.5 mm over a period of 5 min, by 10 μL Hamilton syringe. Control animals received 5 μL of PBS. The burr hole was sealed with bone wax and the incision site was closed with surgical suture. Animals were checked daily for general health (food and water consumption).

### 4.10. Tissue Preparation and Fluorescence Microscopy

After the scheduled time (10 days for FB labeled cells, 25 days for CFSE labeled cells) animals were shortly exposed to CO_2_ and decapitated. Brains were quickly removed and fixed in 4% paraformaldehyde for 48 h, washed with PBS and cryoprotected in 30% sucrose in PBS for at least 72 h. Brains were kept at −80 °C until sectioning on a cryotome. The brains were cut in coronal and sagittal sections 20 μm thick and mounted on Superfrost^®^ glass slides, dried for 12 h at room temperature and stored at −20 °C until staining.

Monoclonal Ki67 antibody was used for determining proliferative potential of inoculated C6 or RC6 cells labeled with CFSE. Tissue slices were allowed to warm to room temperature for 2 h. Then, they were washed with PBS two times. Afterwards, slices were blocked with 2% BSA in PBS for 60 min. Rabbit anti-Ki67 antibody was applied at 1:1000 dilution in 2% BSA/PBS and slices were incubated overnight at 4 °C. After washing with PBS, secondary antibody Alexa Fluor 555 anti-rabbit IgG (H + L) was applied at 1:1000 dilution in 2% BSA/PBS for 120 min at room temperature.

Nuclei were counterstained with PI or Hoechst 33342 for 15 min at room temperature, depending on whether the cells were fluorescently labeled with FB or CFSE and mounted in Mowiol. Tissue slices were examined under Zeiss Axiovert inverted fluorescent microscope equipped with AxioVision 4.8 Software and Leica TCS SP8 confocal microscope (Leica Microsystems, Wetzlar, Germany) equipped with Leica Microsystems LAS AF-TCS SP8 software.

### 4.11. Measurement of Motor Activity

Behavioral testing started 25 days after the surgical procedure, to assess motor activity and learning abilities of the animals. The motor activity of rats was recorded individually for each animal in Opto-Varimex cages (Columbus Instruments, Columbus, OH, USA) that were linked on-line to an IBM-PC compatible computer. The open fields were placed in a light- and sound-attenuated room provided with indirect and homogenous illumination (150 lx in the center of the open field arena). Each cage (44.2 cm × 43.2 cm × 20 cm) was equipped with 15 infrared emitters that were located on the x and y axes. An equivalent number of receivers were located on the opposite walls of the cage. Data were analyzed using Auto-Track software Opto-Varimex-5 (Columbus Instruments). The Auto-Track interface collects data from the Opto-Varimex unit every 1/10th of a second and categorizes the activity. Locomotor activity was defined as a trespass of three consecutive photo-beams. Vertical activity was measured by recording the number of beams that were broken by rearing of the animal. All experiments were performed between 09:00 and 14:00 h. To evaluate intra-session habituation motor activity of the animals was detected for 30 min after the exposure to the open field cages during the testing day. In order to assess inter-session habituation the animals were habituated to the experimental cages for 3 consecutive days [39].

### 4.12. Statistical Analysis

Statistical analysis was performed by GraphPad Prism 6 Software (La Jolla, CA USA). The differences between groups were examined by Student’s *t*-test. Statistical significance was accepted if *p* < 0.05.

Statistical analyses of behavioral data were performed using Statistica 6.0. The data obtained for the habituation sessions during 3 consecutive days of testing were presented as scores for 5 min periods within 30 min registration time (mean values ± SEM, to view time-dependent changes in motor activity during the session—intra-session habituation) and as the summary of activities for the whole registration period (mean values ± SEM, to view inter-session habituation). The number of animals used in the given analysis is specified within the graph bar.

Normality of the data was estimated by Shapiro-Wilk’s test [40]. The data for 5 min scores were analyzed by nonparametric U test, due to extreme non-normality of certain data sets. The data for 30 min scores met the criterion for normal distribution and were analyzed by two-way analysis of variance (ANOVA) with the type of cell line inoculated and time (repeated measure) as factors. Subsequent comparisons were made using the Fisher LSD test. The accepted level of significance was *p* < 0.05.

## Abbreviations

The following abbreviations are used in this manuscript:

GBM	Glioblastoma multiforme
BCNU	1,3-Bis(2-chloroethyl)-1-nitrosourea
TMZ	Temozolomide
MNU	Nitrosourea
ROS	Reactive Oxygen Species
MnSOD	Manganese Superoxid Dismutase
iNOS	Inducible Nitric Oxide Synthase
GPx	Gluthatione Peroxidase
MRP1	Multidrug Resistance-associated Protein 1
MMPs	Matrix Metalloproteinases
DMSO	Dimethyl Sulfoxide
FB	Fast Blue
PI	Propidium Iodide
CAM	Calcein
CFSE	Carboxyfluorescein succinimidyl ester
FBS	Fetal Bovine Serum
CI	Cell Index
BSA	Bovine Serum Albumin
